# An Emerging Role for IQGAP1 in Regulating Protein Traffic

**DOI:** 10.1100/tsw.2010.85

**Published:** 2010-05-18

**Authors:** Mahasin Osman

**Affiliations:** Department of Molecular Pharmacology, Physiology and Biotechnology, Division of Biology and Medicine, Alpert School of Medicine, Brown University, Providence, RI, USA

**Keywords:** IQGAP1, Iqg1p, mTOR, Cdc42, exocyst, protein trafficking, cell proliferation

## Abstract

IQGAP1, an effector of CDC42p GTPase, is a widely conserved, multifunctional protein that bundles F-actin through its N-terminus and binds microtubules through its C-terminus to modulate the cell architecture. It has emerged as a potential oncogene associated with diverse human cancers. Therefore, IQGAP1 has been heavily investigated; regardless, its precise cellular function remains unclear. Work from yeast suggests that IQGAP1 plays an important role in directed cell growth, which is a conserved feature crucial to morphogenesis, division axis, and body plan determination. New evidence suggests a conserved role for IQGAP1 in protein synthesis and membrane traffic, which may help to explain the diversity of its cellular functions. Membrane traffic mediates infections by intracellular pathogens and a range of degenerative human diseases arise from dysfunctions in intracellular traffic; thus, elucidating the mechanisms of cellular traffic will be important in order to understand the basis of a wide range of inherited and acquired human diseases. Recent evidence suggests that IQGAP1 plays its role in cell growth through regulating the conserved mTOR pathway. The mTOR signaling cascade has been implicated in membrane traffic and is activated in nearly all human cancers, but clinical response to the mTOR-specific inhibitor rapamycin has been disappointing. Thus, understanding the regulators of this pathway will be crucial in order to identify predictors of rapamycin sensitivity. In this review, I discuss emerging evidence that supports a potential role of IQGAP1 in regulating membrane traffic via regulating the mTOR pathway.

## MEMBRANE TRAFFIC IS FUNDAMENTAL TO CELLULAR FUNCTIONING

Membrane traffic underlies fundamental cellular functions such as cell growth, division, and cell polarity required for migration, maintenance of tissue organization, and morphogenesis. Newly synthesized proteins are cotranslationally inserted into the endoplasmic reticulum (ER) membrane, properly folded and exported into the Golgi, and sorted in the trans-Golgi networks (TGN) for delivery to endosomes, lysosome secretory granules, and plasma membranes[1,2]. Membrane traffic is mediated by vesicular transport with the actin and the microtubule cytoskeleton serving as tracks, and their associated motor proteins as propellers[3,4,5,6].

Sequencing of the human genome revealed that a range of inherited human diseases arises from dysfunctions in protein traffic[7,8]. Membrane traffic also mediates acquired human diseases caused by infectious agents[9] and it has been implicated recently in the etiology of human cancer[10,11]. Therefore, elucidating the molecular basis of membrane traffic will be crucial for understanding the mechanisms of morphogenesis, and for treatment of degenerative and acquired human diseases ranging from cancer, cystic fibrosis, and Alzheimer's to AIDS and autoimmune disorders. Many of the studies on vesicular traffic have focused on the role of the key regulators, the Rab and the tethering proteins[9,12,13], which represents only the tip of the iceberg in the molecular machineries regulating this multistep and complex cellular process.

## MEMBRANE TRAFFIC AND THE ROLE OF THE ACTIN CYTOSKELETON DYNAMICS

Two types of small GTPases are involved in the regulation of this process. Vesicular movement is regulated by the Rab GTPases[9,14], and the actin cytoskeleton is regulated by the Rho GTPases that regulate membrane protrusions and vesicular traffic mainly by stimulating actin filaments (F-actin) polymerization[3]. In this respect, CDC42 has been shown to regulate retrograde ER>Golgi transport in an N-WASP–dependent manner[15]. Accumulated evidence now enforces the idea that CDC42 regulates post-Golgi traffic in an F-actin–dependent manner[16,17]. Therefore, the actin cytoskeleton dynamics play crucial roles throughout the route from the ER to the plasma membrane, where the tethering proteins, such as the exocyst, play an important role that also requires F-actin polymerization[18].

However, it has long been observed that the vesicles arriving from the inner cytosol have to overcome the physical barrier imposed by the dense actin meshwork underneath the plasma membrane in order to dock on the plasma membrane and prime into the readily releasable state[19]. Recent advancements in microscopy further revealed that dynamic actin remodeling is crucial for exocytosis in two important steps. During docking and tethering of the exocytic vesicle, actin polymerization is required[6]. During a more distal step involving fusion and release, actin meshwork *dis*assembly is required[20,21,22]. The molecular basis of these remodeling events is poorly understood. However, the kinase PKCε has been shown recently to play a critical role in F-actin disassembly following the docking and tethering step of transport vesicles[20,23,24].

Therefore, CDC42 and PKCε are both required for actin remodeling during vesicular traffic. The molecular mechanism(s) underlying their role is still unknown. Evidence from yeast and mammalian cells suggests IQGAP1 as an important player in this process. It plays an essential and conserved role in bundling F-actin[25,26,27], is both an upstream activator and a downstream effector of CDC42, and it regulates protein synthesis and exocytosis[28,29]. Moreover, IQGAP1 appears to be a substrate for PKCε[30].

## IQGAP1 AS A MASTER REGULATOR OF ACTIN DYNAMICS

Mammalian IQGAP1 (Fig. 1A) is a conserved, multidomain, and multifunctional member of a three-isoform family of proteins that differs in tissue distribution and function, and has been the subject of many reviews. It was identified as a target for the Rho GTPases CDC42 and Rac1, but displays no GAP activity towards them[31,32,33,34], in part due to substitution of the catalytic "arginine finger" with a conserved threonine at position 1046 of its RasGAP domain GRD[35]. Further, it interacts with a diverse number of cellular proteins, such as the myosin-essential light chain[36], and integrates signaling networks[37,38]. In many cell types, IQGAP1 localizes to Golgi, the ER, and to the plasma membranes[26,29,33,39]. Despite the evidence that it plays a role as a cortical receptor for microtubule plus ends via CLIP170[39], the current paradigm is that IQGAP1 is the principal regulator of dynamic F-actin assembly at localized cellular sites[40,41]. IQGAP1 promotes actin nucleation and cross-links actin filaments with the Arp2/3 complex

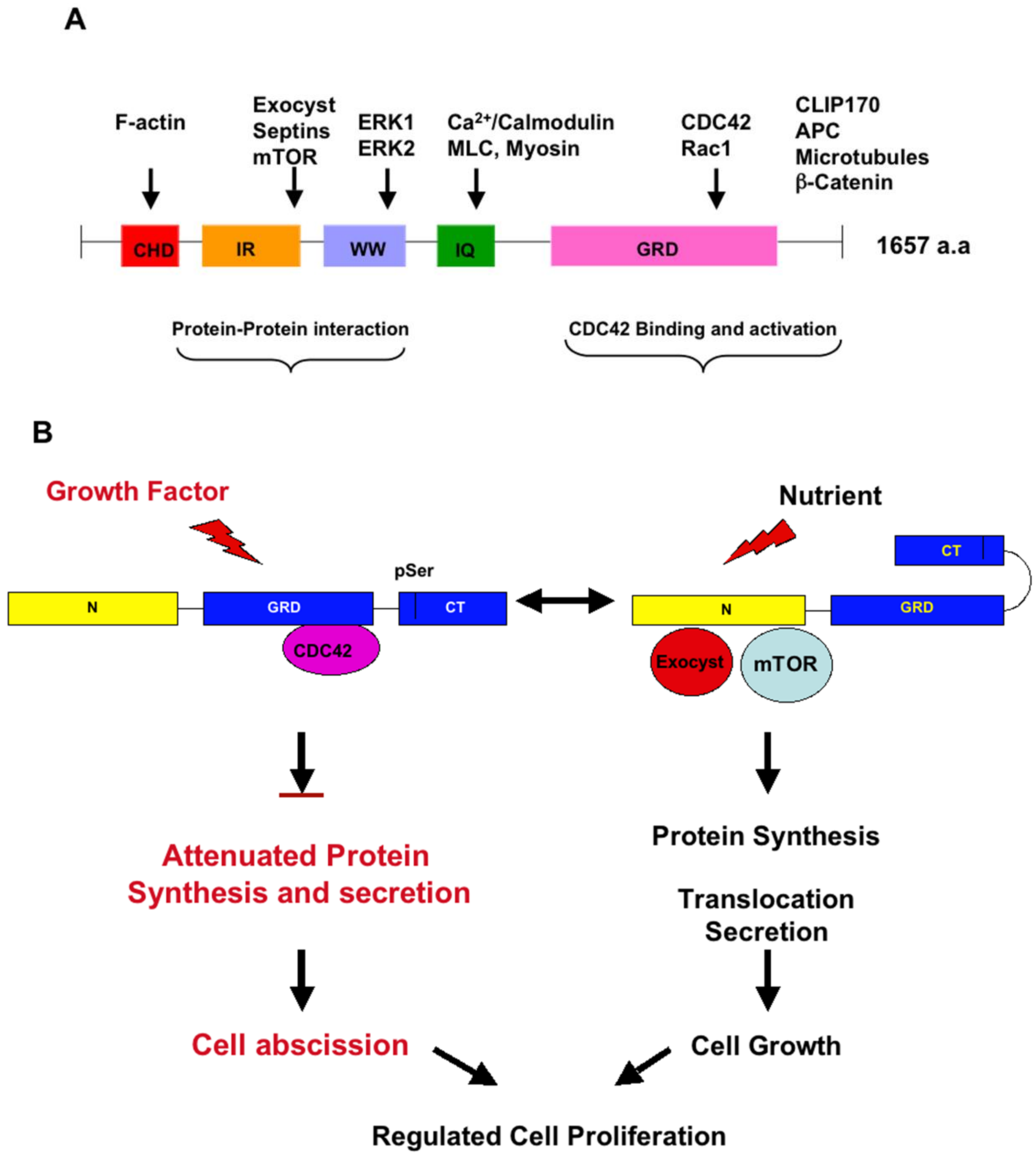

**FIGURE 1.** IQGAP1 is a modular, multifunctional, signaling protein. (A) Schematic representation of IQGAP1 domain structure and a list of some of its binding partners. CHD: *C*alponin *H*omology *D*omain; IR: specific *I*QGAP *R*epeats; WW: proline-rich domain resembling the SH3 interacting domains; IQ: 8 IQ motifs that bind $Ca^{2+}$ calmodulin and myosin; GRD: ras*G*AP-*R*elated *D*omain containing sequences that bind CDC42 and Rac1. (B) A hypothetical model representing the regulatory role of IQGAP1 in protein synthesis and trafficking by which it couples cell growth and division to regulate cell proliferation. IQGAP1 is a conformational switch regulated by phosphorylation and protein-protein interaction (see text for detail). In response to nutrients, IQGAP1 operates in a closed form generated by folding of the C-terminus[29] and interaction with mTOR to promote protein synthesis, and the exocyst to promote secretion, which ultimately results in cell growth. In response to mitogenic signals, IQGAP1 is phosphorylated, leading to binding and activation of CDC42, which in turn results in attenuated secretion and cell growth and promotion of cell division[44].

and N-WASP[26,27]. Significantly, Bloom and colleagues[42] demonstrated that a single monomeric calponin homology domain (CHD) of IQGAP1 could bind F-actin with high affinity. Furthermore,

IQGAP1 interacts specifically with the diaphanous formin mDia1, recruiting it to local sites of F-actin assembly, such as the leading front of migrating cells or the phagocytic cup[41,43]. The range of biological consequences of the IQGAP1 role in F-actin polymerization is yet to be fully appreciated, but it is likely to be key to its role in regulating protein traffic at different sites in the cell.

Upstream regulators of IQGAP1's function in F-actin bundling are yet to be identified, but PKCε is likely to be one. Biochemical evidence demonstrated that PKCε phosphorylates IQGAP1 at the C-terminal serine1443, enhancing its binding to CDC42-GTP during cell scattering[30]. Recent evidence suggested that Ser1443 phosphorylation and binding to CDC42, which attenuates exocytosis[29], are required for IQGAP1 to promote cell migration and transformed phenotypes[44]. Together, these findings suggest that PKCε likely regulates IQGAP1 function in bundling F-actin, as well as in regulating exocytosis, and that these two activities are interconnected. Below, I review the evidence supporting a role for IQGAP1 in regulating membrane traffic.

## IQGAP1 AS A REGULATOR OF PROTEIN TRAFFIC

A role for IQGAP1 in protein traffic was first suggested by work in the budding yeast *Saccharomyces cerevisiae* and followed in different mammalian cell types. In the following two sections, I discuss evidence from yeast and mammals, respectively.

### Yeast IQGAP1, Iqg1p, and Protein Traffic

The budding yeast *S. cerevisiae* buds once per cell cycle using cortical positional cues to mark the division site by initiating a bud early in G1, thereby committing to its division axis[45,46]. Haploid yeast divides in such a manner that a bud is formed next to the previous division site, thus resulting in an axial budding pattern[45]. Genetic analyses have identified Axl1p, Ax2p, Bud3p, Bud4p, and the septins as axial markers for bud site selection[45,47,48]. Null or conditional lethal mutations in each of these genes result in a bipolar budding pattern in the haploid yeast without an effect on the diploid cells[45,47,48]. The positional signal imposed by the bud site selection proteins is decoded and transmitted by the polarity establishment GTPases Bud1p and Cdc42p, which polarize the actin cytoskeleton[49,50,51] and the secretory pathway[52,53] to that site. Fusion of the secretory vesicles at the target site requires a protein complex, the exocyst[51,54,55,56,57], some of its members, such as Sec3p and Sec4p, to localize to the bud tip to promote growth[58,59], then reorient to the mother-bud neck to promote cytokinesis[53]. Sec3p serves as a landmark for localizing the exocyst[54,60]and its polarized localization has been suggested to require the small GTPases Rho1[54] and Cdc42p[61]. Additionally, a recent study demonstrated that the Exo70p subunit of the exocyst serves as a target for both Cdc42 and Rho3 function in polarized exocytosis[62]. A molecular link between the exocyst and the positional markers, and how the exocyst docks to the site of the future bud to promote growth, remain important questions. Similarly, the Cdc42p effector(s) mediating its function in general budding and the mechanism by which it is achieved remain poorly understood.

However, Iqg1p, the ortholog of mammalian IQGAP1, has emerged as an important player in linking targeted exocytosis to directed cell growth and division, i.e., budding. Iqg1p was identified as an effector for the Cdc42p GTPase, and an *IQG1*-null strain displayed phenotypes consistent with a role in polarity, cytokinesis, and protein traffic[25,28,63]. In different strain backgrounds, other groups showed Iqg1p to be essential for cytokinesis via a role in actomyosin ring contraction[64,65], to cooperate with the mitotic exit network[66], and to be regulated by the anaphase-promoting complex[67]. Because the actomyosin ring is dispensable for yeast cell growth and cytokinesis, its role in this process has been challenged[68,69]; thus, Iqg1p must play additional or different roles in the cell.

Indeed, Iqg1p was found to bind the axial marker, Bud4p, and the exocyst landmark, Sec3p, and specifies the bud site as well as promotes cytokinesis in a manner that appears to be directly related to a role in exocytosis and septum deposition[28]. Iqg1 also binds F-actin and its deletion led to disorganization of actin filaments[25], suggesting a role in bundling F-actin similar to its mammalian counterpart. Furthermore, a null strain of Iqg1p exhibited an enlarged vacuole at restrictive temperatures[25], suggesting additional roles in vacuolar-endosomal trafficking. Support for this can be gleaned from the interaction of mammalian IQGAP1 with the conserved endosomal sorting proteins TSG101 and Alix, members of the ESCORT protein complex[70], suggesting a potentially conserved role in endocytosis. Interaction of IQGAP1 with ESCORT proteins may play a role in endosomal sorting as well as in cell abscission, which also requires regulated membrane traffic[28,63,71,72,73,74]. Support for this was provided by the phenotype of *iqg1sec3* double mutant, which exhibited defects in bud site selection and septum deposition[28]. Further work is required for elucidating how and which trafficking pathway does Iqg1p regulate. Nevertheless IQGAP1's role in trafficking appears to be conserved in higher organisms, which I discuss below.

## Mammalian IQGAP1 and Protein Traffic

A similar role for mammalian IQGAP1 in protein traffic has recently become evident and could be gleaned from earlier observations. Several studies showed that IQGAP1 regulates cell-cell junctions in epithelial cells[75,76,77,78], which are considered to be sites for polarized exocytosis[1,2,79,80,81]. It was also shown to influence membrane trafficking in gastric parietal cells[82,83]. Moreover, a negative role for IQGAP1 in secretion has also been reported. Delivery of secretory lysosomes in the immune system requires the clearing of both actin and IQGAP1 from the target site of the plasma membrane[84], and depletion of IQGAP1 by RNAi in mast cells mildly enhanced agonist-stimulated histamine secretion[85]. The mechanism underlying IQGAP1's role in these cellular processes has begun to emerge.

Recent evidence suggests that IQGAP1 is a regulator of membrane traffic. IQGAP1 both positively and negatively regulates exocytosis by regulating CDC42 GTPase. The N-terminal domain of IQGAP1 binds Exo70 of the exocyst[29], the conserved protein complex that mediates the tethering of secretory vesicles to sites of active exocytosis[56,86,87]. Binding of IQGAP1 to CDC42 or constitutive phosphorylation of the C-terminal Ser1443 appears to dissociate the exocyst from the N-terminal domain, attenuates secretion[29], enhances cell migration, and increases cell proliferation[44]. Expression of the N-terminal domain generated a dominant-positive effect on secretion and cell size, whereas expression of the C-terminal domain acted as inhibitor of secretion and promoter of migration and cell proliferation[29,44]. Binding of the exocyst to the IQGAP1 C-terminus has also been reported to enhance cell invasion downstream of RhoA and CDC42[88]. Together, these data support the idea that IQGAP1 regulates secretion by different domains to regulate cellular responses to different signaling cues. A plausible mechanism by which IQGAP1 can achieve these differential roles is by serving as an unconventional GEF/GAP/effector by acting as a conformational switch, itself regulated by phosphorylation and protein-protein interactions (Fig. 1B)[27,29,44]. In this capacity, IQGAP1 may serve as a signaling hub that acts as both an upstream regulator and a downstream effector of converging signaling pathways, which may explain its ubiquitous localization in the cell.

## PERSPECTIVE: HOW IQGAP1 MIGHT REGULATE PROTEIN TRAFFIC

Accumulating evidence predicts that IQGAP1 can potentially regulate protein trafficking at two levels: the level of protein synthesis/translation and the level of docking/tethering by regulating the assembly-disassembly of actin filaments at the exocytic targets. The former may be mediated via binding to mTOR, as discussed below, whereas the latter may occur through binding to the exocyst, which has also been implicated in actin dynamics through the Arp2/3 complex[18]. These roles are likely regulated by CDC42 and PKCε as discussed earlier.

IQGAP1 plays an important role in regulating protein synthesis and translocation[29], perhaps through binding to mTOR[44] and/or translocon subunit Sec61β[29]. Similarly, both yeast and mammalian exocyst have been implicated in translational control through binding to their respective Sec61β translocon subunit[60,89,90]. Significantly, the Sec61β subunit has recently been implicated in membrane traffic to the plasma membrane and the nucleus[91,92], which may indicate a direct connection between protein translation, translocation, and vesicular traffic.

The evolutionarily conserved serine/threonine kinase *t*arget *o*f *r*apamycin (TOR) is known as the center for cell growth control and has been implicated in various aspects of protein traffic. Yeast and mammalian TORs are composed of two distinct *c*omplexes, TORC1 and TORC2 (reviewed in Fingar and Blenis[93], Sabatini[94], Wullschleger et al.[95], and Hall[96]). The mTORC1 is a rapamycin-sensitive complex that regulates accumulation of cell mass by activating mRNA translation and ribosome biogenesis by directly phosphorylating, thereby activating its effector S6K1 (ribosomal S6 kinase 1), a regulator of cell size. The mTORC2 is rapamycin insensitive, but it responds to long-term rapamycin treatment and is considered to act upstream of mTORC1 by directly activating Akt, an effector of PI3K[97]. Akt1, in turn, activates mTORC1 by inhibiting the GAP TSC1/2 complex, thus activating the GTPase Rheb, which activates mTOR. This activation process by the PI3K-Akt is regulated by a negative feedback loop, whereby activated S6K1 inhibits Akt activity by phosphorylating the insulin receptor substrate 1 (IRS-1), thus blocking insulin signaling to Akt[98]. This indicates that mTORC1 and mTORC2 regulate one another during the cell cycle; however, their upstream regulators are not well defined[99].

Recently, mTORC1 has also been shown to affect actin remodeling and protein traffic. Disruption of the mTOR-raptor complex by rapamycin or raptor-siRNA prevented F-actin remodeling and impaired cell motility, through the eukaryotic initiation factor 4E (eIF4E)-binding protein 1 (4E-BP1-5A) subunit, a repressor of protein synthesis[100]. It is unclear whether protein transport to the leading edge of these cells was affected and the role for mTOR pathway in post-Golgi vesicular traffic is only beginning to emerge. It has been suggested that mTOR and Akt1 regulate cell size by influencing the trafficking of surface transporters for nutrients, such as the glucose transporter[101]. Further, hyperactivation of mTOR by down-regulation of TSC1/2 was suggested to disrupt protein trafficking. This was attributed to disruption of microtubule organization as a result of mTOR binding and phosphorylation of CLIP170[102], the microtubule-binding protein that also binds IQGAP1[39]. More recently, mTOR and Akt1 were implicated in TGN to endosome retrograde pathway through an interaction with the Golgi-resident protein GOLPH3[11]. These findings, together with the finding that IQGAP1 interacts with the mTOR pathway[44], provide a platform for elucidating a role for an mTOR-IQGAP1 signaling pathway in regulating post-Golgi traffic and tethering. They further suggest that translation, translocation, and vesicular traffic are likely ordered via a regulatory feedback signaling mechanism in which interplay of IQGAP1-CDC42-PKCε plays an important regulatory role upstream of the mTOR pathway. Exciting times are ahead for elucidating this signaling pathway.

## ACKNOWLEDGMENT

Work in the Osman laboratory was supported by grants from the NIH-NCI (CA104285) and The American Society for Cell Biology (ASCB).
